# Reverse Cholesterol Transport Pathway and Cholesterol Efflux in Diabetic Retinopathy

**DOI:** 10.1155/2021/8746114

**Published:** 2021-10-26

**Authors:** Xinyuan Zhang, Kaiyue Wang, Ling Zhu, Qiyun Wang

**Affiliations:** ^1^Beijing Institute of Ophthalmology, Department of Ophthalmology, Beijing Tongren Hospital, Capital Medical University, China; ^2^Beijing Retinal and Choroidal Vascular Study Group, China; ^3^Save Sight Institute, University of Sydney, Australia

## Abstract

Cholesterol esters, synthesized from cholesterol with long-chain fatty acids, are essential components of plasma lipoproteins and cell membranes that participate in various metabolic processes in the body. Cholesterol can be excreted through the cholesterol reverse transport (RCT) pathway when excessive cholesterol is produced in the extrahepatic cells, which is regulated by the *liver X receptor* (*LXR*) and its downstream regulators *ATP-binding cassette subfamily A member 1* (*ABCA1*) and *ATP-binding cassette subfamily G member 1* (*ABCG1*) genes. Abnormal cholesterol metabolism is closely associated with the development of diabetic retinopathy (DR). However, the precise underlying mechanism of the RCT pathway in the pathogenesis of DR is still not fully understood. This review focused on cholesterol metabolism, with a particular emphasis on the RCT pathway and its correlation with the development of DR. Particular attention has been paid to the key regulators of the RCT pathway: *LXR*, *ABCA1*, and *ABCG1* genes and their potential therapeutic targets in the management of DR.

## 1. Introduction

The association between abnormal lipid metabolism and microvascular complications of diabetes has received considerable attention recently. The imbalance of the reverse transport (RCT) pathway of cholesterol under high glucose pathological conditions is a new focus of research becoming a hot topic. This review summarizes the recent progress on cholesterol metabolism abnormalities, particularly emphasizing the association of the RCT pathway in the pathogenesis of diabetic retinopathy (DR). This novel research direction may lead to an insight into discovering biomarkers and molecular targets for tailored therapies. The key regulators of the RCT pathway will expect to be a frontline in the prevention and treatment of DR.

## 2. Cholesterol: Biosynthesis and Regulation

### 2.1. Synthesis and Transportation

Cholesterol is the most common and abundant molecule in the body, comprising approximately 30% of all mammalian cell membranes [1]. In addition to supporting the structure of cells, cholesterol is the precursor of synthetic bile acids, vitamin D, and steroid hormones [2]. Cholesterol further exhibits essential physiological roles in cell transport, signal transduction, and nerve conduction [3, 4]. Exogenous cholesterol is mainly derived from dietary sources, as well as from internal endogenous biosynthetic pathways. The liver and intestinal mucosa are the main organs for cholesterol synthesis, in addition to the brain.

Metabolism of cholesterol includes two main pathways: endogenous synthesis and exogenous absorption. Almost all mammalian cells can synthesize cholesterol, and the most active site of synthesis is the liver. Acetyl-CoA is used in cells to synthesize cholesterol de novo through a multistep enzymatic reaction. Acetyl-CoA reductase is the key enzyme that catalyzes mevalonate production from Acetyl-CoA. Endogenous cholesterol synthesized in hepatocytes is mainly transported by low-density lipoprotein (LDL) to the peripheral tissues in the form of LDL cholesterol (LDL-C). LDL-C found in the blood is recognized by the LDL receptor and transported into the cells [5]. The second pathway involves the exogenous cholesterol pathway: the small intestine directly obtains exogenous cholesterol from dietary sources, which mainly occurs in the upper and middle parts of the small intestine. Niemann-Pick C1-Like protein (a polytopic transmembrane protein of 1332 amino acids) mediates cholesterol absorption and is highly specific and inhibits plant sterol uptake [6].

### 2.2. Conversion and Efflux

The metabolic pathway of cholesterol includes conversion and efflux. Cholesterol synthesized in the liver is transported to peripheral tissues through circulation to meet the body's regular metabolic needs. Cholesterol is not entirely oxidized and decomposed into CO_2_ and H_2_O in mammalian cells. Cholesterol is subsequently converted to other compounds containing a cyclopentane polyhydrophenanthrene nucleus *via* enzyme catalysis (oxidation and reduction reactions). Under normal circumstances, excessive cholesterol is excreted from cells and transported to the liver through circulation and is metabolized in hepatocytes and finally excreted through bile and feces. Such a process is called reverse cholesterol transport (RCT).

Cholesterol efflux from cells has been proved to be an early step of RCT. The RCT pathway plays a vital role in maintaining the kinetic balance of cholesterol metabolism and is regulated by several genes and proteins. Liver X receptor (LXR) and ATP-binding cassette transporters A1 (ABCA1) and G1 (ABCG1) have been identified as the key regulators in atherosclerosis [7]. It has been reported that serum capacity to induce ABCA1- and SR-BI-mediated cholesterol efflux is impaired in diabetic patients with incipient or overt nephropathy. Such impairment may contribute to the accelerated development of atherosclerosis in these patients, suggesting that the capacity of serum to induce cellular cholesterol efflux is an independent predictor of atherosclerosis and diabetic nephropathy [8].

### 2.3. Liver X Receptors (LXRs) and Metabolism of Cholesterol

#### 2.3.1. Classification and Distribution of LXR

LXR, a ligand-activated nuclear transcription factor, is a member of the nuclear receptor superfamily and is involved in regulating cholesterol metabolism. LXR is a receptor for cholesterol metabolism and lipid biosynthesis. As an insulin sensitizer, LXR has been found to upregulate glucose transporter type 4 (GLUT4) expression. Furthermore, LXR agonist promotes glucose uptake in adipocyte, suggesting that activation of LXR could alter the expression of genes in liver and adipose tissue, limiting glucose output and improving peripheral glucose uptake [9]. In addition, LXR participates in water-electrolyte balance and immune response, regulating the physiological functions of a variety of apolipoproteins (apos) [10], thereby maintaining cholesterol homeostasis [10].Two subtypes of LXR in humans have been identified: LXR*α* (NR1H3) and LXR*β* (NR1H2). LXR*α* is mainly expressed in the macrophages from the liver, kidney, adrenal gland, and small intestine, while LXR*β* is almost expressed in all of the tissues throughout the body [11]. LXR*α* and LXR*β* share over 75% amino acid sequence identity and are important regulators in the cholesterol and lipid metabolism process. Balasubramanian et al. found that LXR*α* has a significant role in regulating the expression of GLUT4 in adipocytes and potential targets in treating diabetes [12]. LXR*α*(-/-), LXR*β*(-/-), and LXR*α*/*β*(-/-) mice developed acellular capillaries and endothelial progenitor cell dysfunction similar to the streptozotocin-injected DBA/2J mice fed a high-fat Western diet, but activation of LXR with a synthetic ligand reversed those pathological changes [13].

**Figure 1:**
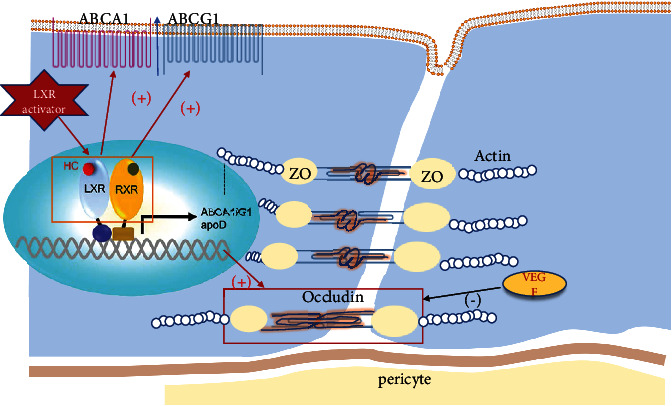
Liver X receptor perturbations of lipid metabolism in diabetic retinopathy. Under normal physiological conditions, transcriptional activity of LXRs is increased in response to elevated cellular levels of cholesterol. A high glucose environment attenuates activation of LXR, inducing the downregulations of transporters ABCA1 and ABCG1 on the cell membrane. Consequence of cellular cholesterol accumulation causes oxidative stress and inflammation in vascular endothelial cells. Furthermore, the increased expression level of VEGF downregulates tight junction proteins ZO-1 and occludin, leading to blood-retinal breakdown. LXR agonists can eventually reverse this pathological process. LXR: liver X receptor; RXR: retinoid X receptor; ABCA1: ATP-binding cassette subfamily A member 1; ABCG1: ATP-binding cassette subfamily G member 1; apo: apolipoprotein; ZO-1: zonula occludens-1.

LXR combines with their binding partner retinoid X receptor (RXR) in the nucleus to form an LXR/RXR heterodimer. Upon LXR agonist binding, the LXR/RXR heterodimer releases a corepressor that possesses transcription factor activity and is bound to the target gene-specific gene starter sequence. Such transcription plays a major role in regulating the absorption, storage, excretion, and metabolism of cholesterol [14, 15] (Figure 1).

Endogenous agonists of LRX are mainly derivatives of cholesterol, including hydroxysterols 24(S),25-epoxycholesterol, 22(R)–hydroxycholesterol, and 24(S)-hydroxycholesterol. Exogenous agonists are mainly synthesized, such as T0901317 and GW3965. The majority of these exogenous agonists are nonselective.

#### 2.3.2. Target Genes of LXR associated with Cholesterol Metabolism

Activation of LXR initiates the transcription of multiple genes, participates in the RCT pathway, maintains lipid metabolism balance, and promotes cholesterol outflow. The direct target genes of LXR which have been identified include *ABCA1* [16], *ABCD2*, *ATP-binding cassette subfamily G member 1* (*ABCG1*) [17], *SREBP-1c* [18], *FAS* [19], *CETP*, *LPL*, *CYP7a* [20], *apoE* [21], and *GLUT4* [22]. The main downstream regulators are *ABCA1*, *ABCG1*, *SREBP-1c*, *apoE* genes, and phospholipid transporter [23]. ATP-binding cassette transporters (ABC transporters) are a type of one-way transmembrane transporter, which uses ATP as an energy source. ABC transporters mediate the transmembrane transport of several different substrates, such as amino acids, lipids, ions, and sugars. The target genes of LXR mainly include the four ABC transporters, namely, *ABCA1*, *ABCG1*, *ABCG5*, and *ABCG8* [23, 24], all of which are involved in cholesterol transport. Binding of ligand to LXR results in the upregulation of ABCA1 and ABCG1 in cells [25–27].

ABCA1 contains an asymmetric transmembrane domain and is composed of a dimer containing six transmembrane segments. ABCA1 is widely expressed on a variety of cell membranes and binds to apolipoproteins during RCT and participates in HDL formation. ABCA1 promotes the redistribution of cholesterol and sphingomyelin and assists free cholesterol flow to lean apolipoproteins, which are esterified into mature HDL particles [28]. ABCG1 is a semitransporter that mainly transfers intracellular cholesterol to extracellular mature HDL, thereby reducing intracellular cholesterol concentration [29, 30]. ABCG1 mediates intracellular cholesterol transfer to extracellular mature HDL which is the main pathway for cholesterol efflux from endothelial cells, concomitant with a higher level of ABCG1 than ABCA1, which is activated by LXR [31]. In addition, ABCG1 plays a significant role in mediating cholesterol efflux and HDL transport, preventing cellular lipid accumulation in endothelial cells [32, 33]. HDL combines free cholesterol excreted from cells (mainly macrophages and endothelial cells) and promotes its transport to the liver under the action of cholesterol ester transfer protein (CETP). This causes the formation of bile acids and their excretion or the production of hormones by cholesterol in tissues. LXR mediates HDL return to the liver via directly acting on CETP [34]. Furthermore, HDL involves in the RCT pathway through lipid exchange with other apolipoproteins.

Immunochemistry confirms that both ABCA1 and ABCG1 are localized to the ganglion cell layer, outer plexiform layer, and RPE in the retina from primates, suggesting that RCT is an HDL-based intraretinal lipid transport. ABCA1 seems to localize more intensively to the apical side of the RPE, while ABCG1 localizes to the basal side. ABCA1 or ABCG1 is 1.4- or 2.5-folds greater in the human retina than in the liver [35]. Moreover, ABCA1, localized on both sides of the retinal epithelial cells, is modulated by LXR *in vitro* [36, 37]. However, it remains to be confirmed that the inducer LXR and the impaired ABCA1 and/or ABCG1 contribute to lipid efflux and subsequently lead to DR.

#### 2.3.3. LXR and Apolipoprotein Profiles

Blood lipids, including triglycerides, cholesterol, and lipids (phospholipids, glycolipids, and steroids), are insoluble in water and are mainly transported in the form of lipoproteins. Lipoproteins are mainly classified into chylomicrons (CM), very low-density lipoprotein (VLDL), low-density lipoprotein (LDL), and high-density lipoprotein (HDL) particles. The main function of HDL is to participate in the reverse transport of cholesterol. The protein part of the plasma lipoproteins is called apolipoprotein, which is divided into the following five types and several subclasses: apolipoprotein A (apoA1, apoA2, apoA4, and apoA5), apoB (apoB48 and apoB100), apoC (apoC-I, apoC-II, apoC-III, and apoC-IV), apoD, apoE, apoF, apoH, apoL, apoM, and apolipoprotein (a).

Different lipoproteins contain different apolipoproteins that carry lipids and stabilize the lipoprotein structure. Also, certain apolipoproteins possess key enzyme activities that regulate lipoprotein metabolism and participate in lipoprotein receptor recognition. LXR is considered the key regulatory target of lipid metabolism and can directly or indirectly regulate *apo* mRNA expression, protein synthesis and secretion, and apolipoprotein-mediated cholesterol outflow [38, 39]. Following LXR activation by an agonist, cholesterol efflux is promoted through the RCT pathway and is taken up by apoA-I and apoE to form new HDL molecules, which enter the visceral liver for further transformation and/or decomposition [40].

apoA-I is mainly secreted by the liver and small intestine and is the main structural protein and responsible for the major functions of HDL. apoA-I, a 28 kDa single-chain polypeptide consisting of 243 amino acid residues, possess antioxidant, anti-inflammatory, and antiatherosclerotic activity [41]. apoA-I participates in intracellular cholesterol efflux with ABCA1 [42] to transport blood cholesterol to the liver [43]. apoA4 is a major protein component of lymph chylomicrons, VLDL, and HDL. Both apoA-I and apoA4, essential for lipoprotein metabolism to maintain plasma lipid levels, are modulators of vascular disease [44].

apoA1 can be upregulated by LXR dependent or independent of ABCA1. Endogenous ABC1 gene expression and apolipoprotein A1-mediated cholesterol efflux are upregulated via both receptor ligands, indicating that activation of the LXR-RXR heterodimer by ligands contributes to the ABCA1 pathway therapeutic modulation [9]. The LXR agonist GW3965 has also been shown to increase apoA1 expression independent of ABCA1; apoA1 participates in brain-blood barrier regulation and serves to integrate peripheral and CAN lipid metabolism [25].

apoB is a major component protein of LDL, very low-density lipoprotein (VLDL), intermediate-density lipoprotein (IDL), chylomicrons, and LDL. apoB plays a significant role in lipoprotein transport. Plasma levels of apoB and LDL were increased by IDOL (the E3 ubiquitin ligase) which was induced by a synthetic LXR agonist in a nonhuman primate model [45]. Intracellular trafficking of the apical apolipoprotein B was also downregulated by LXR agonists to lower the risk of cardiovascular disorders and T2DM in an *in vitro* model [46].

Liver secreted apoE in lipid metabolism after entering circulation. apoE is also mainly synthesized in the astrocytes in the brain. apoE has three major allies: *apoE-ε2* (cys112, cys158), *apoE-ε3* (cys112, arg158), and *apo-ε4* (arg112, arg158). *apoE-ε2* has both increased and decreased risk for atherosclerosis [47]. *apoE-ε3* has been thought to be the “neutral” *apoE* genotype; *apo-ε4* was reported to be involved in the pathogenesis of atherosclerosis [47] and Alzheimer's diseases [48]. LXR directly regulates *apoE* gene expression in macrophages to control its expression [38]. apoE plays an antiatherosclerotic effect by cooperating with specific apolipoproteins, maybe *via* maintaining the stability of ABCA1. apoE4 is an independent risk factor for Alzheimer's disease [49].

apoD in the formation of HDL is positively regulated by LXR *via* reverse transport of cholesterol by promoting cholesterol esterification. apoM is mainly synthesized by the liver and kidneys to regulate the formation of pre-*β*-HDL *via* reverse cholesterol transport [50]. apoM also modulates the expression of inflammatory factors and adhesion molecules [49].

Although apoC-II and apoC-III have been implicated in cardiovascular diseases and DM, few literatures reported their link to the RCT pathway. It is interesting to investigate the correlations between other apo members and LXR in future studies.

#### 2.3.4. LXR and Cholesterol Transport under Normal Physiological Conditions

LXR activation promotes cholesterol efflux in cells. In the presence of excess intracellular cholesterol, cholesterol oxidation derivatives activate the LXR/RXR heterodimer, initiate the target gene transcription, increase the protein expression of ABCA1 and ABCG1 on the cell membrane, and promote the excretion of cholesterol from the cell. In addition, LXR indirectly inhibits cholesterol synthesis and small intestine absorption *via* activating the transcriptional expression of its downstream genes. Cholesterol is synthesized in hepatocytes using Acetyl-CoA. This process is inversely regulated by the RCT pathway *via* inhibiting the expression of the key enzyme squalene synthase [51]. Following LXR activation, *ABCA1*, *ABCG5*, and *ABCG8* are upregulated to reduce cholesterol absorption in the small intestine. In addition, LXR activation also promotes liver cholesterol conversion and efflux to maintain the whole-body cholesterol level because the liver is the main organ to be used for cholesterol excretion. Cholesterol is converted into bile acids and excreted in the liver using cholesterol 7*α* hydroxylase (CY7PA). Activated LXR upregulates the expression levels of ABCG5 and ABCG8 and promotes the secretion and/or excretion of hepatobiliary cholesterol.

## 3. Cholesterol Metabolism in the Retina

Cholesterol metabolism in the retina includes uptake from the systemic circulation, self-synthesis, and clearance. However, the underlying mechanism is not fully understood. The blood-retinal barrier and blood-brain barrier are the unique structures that represent a functional interface between the bloodstream and neuronal vascular microenvironment, respectively. Cholesterol derived from the brain and retina is mainly found in the form of nonesterified cholesterol, suggesting that the retina may maintain cholesterol metabolism balance in a similar way to that of the brain. Current research indicates that the cholesterol in the brain all relies on its own synthesis and that cholesterol is not obtained from systemic circulation [52]. Previous studies have rarely examined cholesterol metabolism in the retina, and very little is known regarding the balance of cholesterol metabolism in the neuroretina. Cholesterol is found to be synthesized in the retina using radioactive cholesterol precursors and immunohistochemically labelled cholesterol synthesis rate-limiting enzyme HMGCR in rats [53]. Specific compounds were shown to inhibit the last step of cholesterol synthesis which leads to degradation of 7-dehydrocholesterol, suggesting that a large amount of cholesterol precursors is accumulated in the retina [54]. Furthermore, reduction of the cholesterol levels is found to be accompanied by progressive retinal degeneration, suggesting that the retinal tissue requires cholesterol metabolism balance *via* endogenous cholesterol synthesis in order to maintain the normal structure and function of the retina.However, a controversial finding is also reported, showing that cholesterol synthesis does not occur in the retina by itself but from the systemic circulation. Cholesterol enters the retinal neuroepithelial layer through the RPE layer. Fluorescently labelled cholesterol was detected in the RPE layer, and a small amount of fluorescence is presented in the neural retina following injection of fluorescently labelled low-density lipoproteins in the rhesus monkeys [55]. The strong signal of fluorescently labelled LDL is detected in the RPE layer of the retina and then is found to be gradually spread in the entire retina [56]. Only a small amount of fluorescent label is detected in the HDL cholesterol group. Such finding further illustrates that RPE and neural retinas could rapidly take up cholesterol from the systemic circulation, suggesting that LDL may be an important transporter that uptakes systemic cholesterol to the retina.

## 4. Correlation between Cholesterol Metabolism, Diabetes, and Diabetic Retinopathy

Elevated serum LDL-C and reduced HDL-C levels are high-risk factors for cardiovascular disease, whereas it has also been shown that excessive cholesterol concentration leads to hypercholesterolemia. Dyslipidemia with microvascular complications has been intensively investigated [57]. However, the associations between traditional lipid markers and DR remain controversial. The full understanding and recognition of the importance of lipid-lowering treatment are considered important for the tertiary prevention of diabetes microvascular complications [58, 59].

The mechanism of dyslipidemia promotion may be at several certain levels. The retinal-specific lipid metabolism contributes to low-grade inflammation resulting in diabetic BRB breakdown [60]. Furthermore, the number of bone marrow-derived (BMD) circulating angiogenic cells (CACs) is decreased in dyslipidemia. Action to Control Cardiovascular Risk in Diabetes found the associations between blood lipid levels and hard retinal exudation [61–63]. The total serum cholesterol or the LDL-C of the patients is increased at baseline, and retinal hard exudation is more likely to occur in the Early Treatment Diabetic Retinopathy Study (ETDRS) [64]. Klein and colleagues evaluate the correlations between the serum level of blood lipids in a five-year followed up cohort. A correlation between DR and the total cholesterol/HDL-C ratio is found, confirming that cholesterol-lowering treatment may delay DR progression [65]. The Hoorn study [66] evaluates the risk factors for DR in 2484 Caucasians aged between 50 and 74 years. The results demonstrate that the prevalence of DR is positively associated with body mass index (BMI), serum cholesterol, and triglyceride levels. In addition, elevated levels of plasma total cholesterol and LDL-C are associated with the occurrence and development of retinal hard exudation. To evaluate the risk factors for diabetic microvascular and macrovascular complications, Nazimek-Siewniak and colleagues [67] investigate the associations between the serum levels of fasting blood glucose, total cholesterol, triglycerides, blood pressure, and BMI with the development of DR in 2175 newly diagnosed type 2 diabetes patients. It is found that elevated total cholesterol significantly increased the risk of developing proliferative retinopathy in diabetic patients. Hadjadj et al. report that high triglycerides are independent predictors of renal and retinal complications in patients with type 1 diabetes without end-stage renal disease [68].

It is further reported by the Diabetes Control and Complications Trial (DCCT) that the total HDL-C ratio and LDL-C concentration are predictors for the development of CSME and hard exudation. Hyperlipidemia may increase the risk of CSME and hard retinal exudation, whereas lipid-lowering treatment in patients with type 1 diabetes may reduce the risk of CSME [69]. On the other hand, LDL is found to be an independent risk factor for DR [70], which is in line with the findings compared with simvastatin alone. HDL-C correlates negatively with the occurrence of DR, PDR, and DME in a cohort study in patients with T2DM [71].

Although traditional lipid profiles are found to be associated with the pathogenesis of DR, the conclusive link is still controversial. The correlation between the serum level of total cholesterol and HDL with DR and retinal hard exudative was not found associated with the serum level of total cholesterol and HDL by the Wisconsin Epidemiological Study of Diabetic Retinopathy (WESDR) [72]. The FIELD study [54] enrolled a total of 9995 T2DM patients aged from 50 to 75 years. The patients are followed up for 5 years to evaluate whether long-term use of fenofibrate, the peroxisome proliferator-activated receptor alpha agonist lipid-lowering therapy, could reduce DR progression and the number of laser treatments. The participants receiving fenofibrate (200 mg/d) exhibited a 31% reduction of reduced incidence of DR or progressed to DME in the first laser treatment requirements compared with the placebo group. The Action to Control Cardiovascular Risk in Diabetes (ACCORD) trial is a randomized, multicenter study. Among the 10,251 middle-aged and older participants with T2MD, a subgroup of 2856 subjects were evaluated for the effects of the intensive or standard treatment for glycemia, dyslipidemia, or systolic blood-pressure control at 4 years on the progression of DR. The results showed that at 4 years, the rate of progression of DR was 6.5% with fenofibrate in comparison with 10.2% of the placebo [73]. However, no conclusive relationship between the lipid effects of fenofibrate and the presence or progression of DR was found in both FIELD and ACCORD studies; Wong et al. suggested that the beneficial effects of fenofibrate on DR may be through regulating the levels of apos [74]. Hadjadj et al. also found that apoA1 is correlated negatively with DR, while apoB and apoB/apoA1 ratios are correlated positively with DR [68], which is consistent with others, showing that serum apoB and apoB/apoA ratios are the most important risk factors for PDR and CSME [59].In summary, current guidelines suggest that the use of lipid-lowering drugs as adjuvant therapy can control diabetes-related microvascular complications [58, 75]. However, in certain large epidemiological studies, such as DCCT, EDIC, and WESDR, there is no direct association between the progression of DR and the levels of peripheral blood lipids. It is speculated that retinal-specific mechanisms may play an important role in the progression of DR caused by cholesterol [76], which remains to be clarified in the future. Although several researchers suggest that LDL, HDL, and apo are related to DR in varying degrees, no clear markers exist for the prediction of the occurrence and development of DR. Further research on the association between dyslipidemia and DR could provide additional evidence for the prevention of the development DR. More recently, the application of the nontraditional lipid marker apo has been continuously explored in the detection of lipid-associated disease, and it is expected that further breakthroughs will be achieved in the near future.

## 5. LXR and Diabetic Retinopathy

LXR is not only involved in the regulation of lipid metabolism but also considered a part of the insulin signaling pathway, which is involved in the process of glucose metabolism [22]. Activation of LXR regulates the systemic inflammatory response by promoting cholesterol metabolism and inhibiting the inflammatory response. LXR*β* polymorphisms with type 2 diabetes mellitus and obesity are also evidenced recently [77]. Figure 1 summarizes the effects of the LXR pathway and LXR agonist in the pathogenesis of DR.

**Figure 2:**
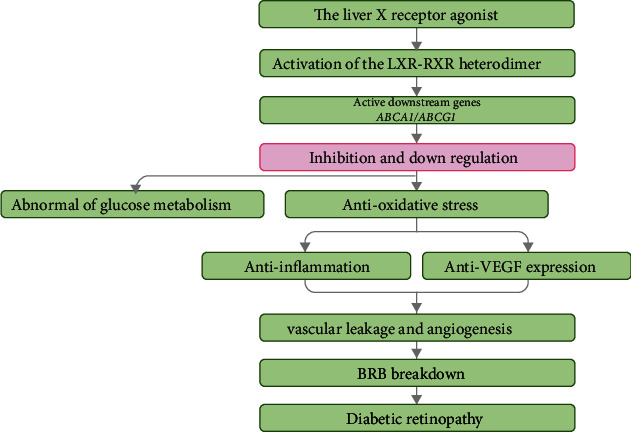
The liver X receptor agonist and diabetic retinopathy. This flow diagram illustrates the regulatory mechanism of activation of LXR signaling by the LXR agonist in DR. LXR: liver X receptor; BRB: blood-retinal breakdown; RXR: retinoid X receptor; DR: diabetic retinopathy.

It is speculated that LXR may participate in the development of DR through the following mechanisms (Figure 2).

### 5.1. Regulation of Glucose Metabolism

LXR participates in the regulation of lipid metabolism and acts as an insulin sensitizer, playing a significant role in glucose metabolism. LXR can be activated with glucose in the liver, which is an endogenous ligand of LXR [78]. Furthermore, LXR inhibits endogenous glucose production, converts excess glucose to glycogen or fatty acids, and stores these molecules in the adipose tissue [79]. Glucose transporter GLUT4 plays a key role in insulin-mediated glucose uptake in adipose tissues. Impairment of GLUT4 is associated with obesity and type 2 diabetes. The promoter of GLUT4 is a direct target gene of LXR. LXR agonists upregulate GLUT4 in adipocytes and promote peripheral glucose uptake [9]. In animal models of diabetes, the LXR agonists GW-3965 and T0901317 significantly reduce blood glucose levels in hyperglycemic mice, improving insulin sensitivity, inhibiting the hepatic synthesis of glycogen, and reducing glucose output [80, 81]. In summary, as a transcription switch, LXR integrates glucose metabolism and lipid metabolism and may be involved in the development of DR.

### 5.2. Anti-inflammation

Inflammation and oxidative stress are considered important pathological mechanisms of DR. Increased retinal or systemic inflammatory factors in DR patients or animals are reported. Oxidative stress is responsible for the inhibition of inflammation and oxidative stress, which prevents the development of DR significantly [82, 83]. Because activation of LXR inhibits inflammatory response *via* promoting cholesterol metabolism, LXR is considered to be one of the key receptors that regulate systemic inflammation [13, 84]. Anti-inflammatory mechanisms of LXR include transrepression, posttranscriptional level regulation, and inhibition of inflammatory factor expression via regulating lipid metabolism. Activation of LXR inhibits the activity of transcription factors, such as NF-*κ*B, to block NF-*κ*B-dependent inflammatory factors [85], regulating the expression of inflammatory factors via posttranscriptional mechanisms. Activation of LXR further inhibits the expression levels of the inflammatory factors *via* regulating lipid metabolism [86]. This is supported by the finding that oral administration of LXR agonists significantly prevents DR development and reduces inflammatory cells in the retina via downregulating oxidative stress genes in an STZ-induced diabetic model [13]. LXR is also found to normalize reverse cholesterol transport and prevent diabetes-induced inflammation in retinal cells. Activation of LXR reduces the number of proinflammatory macrophages and prevented DR-like pathology. The aforementioned findings suggest that LXR agonists may prevent diabetic vascular disease by inhibiting the inflammatory response and oxidative stress.

### 5.3. Inhibition of Angiogenesis

Vascular endothelial growth factor (VEGF) is a specific mitogenic protein secreted by endothelial cells, with the VEGF family of VEGF-A, B, C, D, and E and placental growth factor (PlGF). These VEGFs activate the downstream cascade after binding to VEGF receptors (VEGFR-1, VEGFR-2, and VEGFR-3).

VEGF-A is the most abundant and biologically active form among the VEGF family in tissues. Upregulation of VEGF in retinal vascular endothelial cells leads to an increase in the permeability of capillaries, which subsequently breaks down the blood-retinal barrier and acceleration of neovascularization and eventually exacerbates the progression of DR [87].

LXR agonists improve cholesterol metabolism and inhibit human umbilical vein vascular endothelial cell proliferation and migration, as well as formation of luminal structures *via* downregulating VEGF2 l [88]. Activation of the LXR receptor is found to suppress angiogenesis via induction of apoD in human umbilical vein endothelial cells [89]. It is also found that LXR activation reduces angiogenesis by impairing lipid raft localization and signaling of VEGF receptor 2. Furthermore, VEGF-A can be upregulated in the presence of LDL, suggesting that VEGF-A synergizes the effects of LDL contributing to the progression of atherosclerosis [90]. Therefore, further exploration of the association between VEGF expression, LXR pathway, and cholesterol metabolism may aid the identification of new targets for DR intervention.

## 6. LXR Agonist Modulates Diabetic Retinopathy Pathophysiology

LXR has been proved to be the novel therapeutic target in both animal models and humans. Synthetic oxysterol as the gene-selective LXR modulator mediates transcriptional activation of ABCA1 gene expression with minimal effects on SREBP-1c both in vitro and in vivo in mice [91]. Selective LXR*α* and LXR*β* increased HDL cholesterol and increased liver triglycerides in wild-type mice, induced ABCA1 expression, and stimulated cholesterol efflux in macrophages from both LXR*α*- and LXR*β*-deficient mice [92].

Synthetic chemical agonists of LXT have been shown efficacious for inhibition of DR in diabetic animal models. However, due to the adverse effect profile, including hypertriglyceridemia and hepatic steatosis, the trials were halted [93, 94]. N,N-Dimethyl-3*β*-hydroxy-cholenamide (DMHCA) is a synthetic oxysterol with shortened sidechain and amide moiety which exhibits only limited activity for increasing hepatic SREBP-1c mRNA and does not stimulate triglyceride synthesis and alter circulating plasma triglycerides in comparison with known nonsteroidal LXR agonists [95]. Cell-based studies also indicate that this selective modulator inhibits cholesterol accumulation. DMHCA has been shown to rescue retinal and BM dysfunction in diabetes, thereby restoring retinal structure, function, and cholesterol homeostasis [95]. DMHCA has been tested in patients with advanced solid malignancies and lymphoma and with refractory malignancies [96].

There are also several ongoing clinical trials of DMHCA targeting on DR. An observational study is being carried out to investigate if LXR activation can restore cholesterol homeostasis in the diabetic retina and rescue diabetes-induced bone marrow dysfunction to sustain circulating angiogenic cells (CAC) and macrophages (ClinicalTrials.gov Identifier: NCT03403686).

## 7. Perspective

Previous studies have confirmed an inextricable link between abnormal cholesterol metabolism and the development of DR. Further research on lipid metabolites, such as the identification of cholesterol and nontraditional lipid markers, will aid the clarification of their role in disease development and provide new intervention directions for the preventive diagnosis and treatment of DR. All of this is expected to become a novel research hotspot with regard to the prevention and treatment of DR.

In summary, the current literature suggests that the RCT pathway plays an important role in the process of cholesterol metabolism. Recently, several studies have examined the interaction of LXR thoroughly with its agonists in an attempt to provide potential directions for clinical transformation. At present, a variety of artificial LXR agonists have been synthesized. However, since LXR agonists are applied to the whole body, they may upregulate SREBP-1c expression and produce related undesired adverse reactions, including induction of adipogenesis, hypertriglyceridemia, and liver steatosis. Although there are no safe and effective LXR agonists for clinical application yet, the development of LXR selective receptor agonists might promote the identification of novel LXR agonists that do not alter liver and plasma triglyceride levels. LXR agonists could be tailored therapeutical targets in the treatment of DR and other diseases with abnormal cholesterol metabolism in the near future.

## Data Availability

This review did not include any original data.

## Abbreviations

ABCA1: ATP-binding cassette subfamily A member 1
ABCG1: ATP-binding cassette subfamily G member 1
ACCORD: Action to Control Cardiovascular Risk in Diabetes
Apo: Apolipoproteins
BMD: Bone marrow-derived
BMI: Body mass index
CAC: Circulating angiogenic cells
CETP: Cholesterol ester transfer protein
CM: Chylomicrons
CSME: Clinical significant macular edema
CY7PA: Cholesterol 7*α* hydroxylase
DMHCA: N-Dimethyl-3*β*-hydroxy-cholenamide
DR: Diabetic retinopathy
ETDRS: Early Treatment Diabetic Retinopathy Study
GLUT4: Glucose transporter type 4
HDL: High-density lipoprotein
LDL: Low-density lipoprotein
LXR: Liver X receptor
PlGF: Placental growth factor
RCT: Cholesterol reverse transport
RXR: Retinoid X receptor
VEGF: Vascular endothelial growth factor
VEGFR: VEGF receptors
VLDL: Very low-density lipoprotein
WESDR: Wisconsin Epidemiological Study of Diabetic Retinopathy.
